# Costimulators expressed on human endothelial cells modulate antigen-dependent recruitment of circulating T lymphocytes

**DOI:** 10.3389/fimmu.2022.1016361

**Published:** 2022-10-06

**Authors:** Thomas D. Manes, Vivian Wang, Jordan S. Pober

**Affiliations:** Department of Immunobiology, Yale School of Medicine, New Haven, CT, United States

**Keywords:** T cell, transendothelial and transepithelial migration, costimulating molecules, cytokine expression, flow chamber assay

## Abstract

Endothelial cells (ECs) can present antigens to circulating effector memory T cells (T_EM_) and to regulatory T cells (T regs), triggering antigen-specific extravasation at specific sites where foreign antigens are introduced, e.g. by infection or transplantation. We model human antigen-induced transendothelial migration (TEM) using presentation of superantigen by cultured human dermal microvascular (HDM)ECs to isolated resting human peripheral blood T cell subpopulations or to T effector cells activated *in vitro*. T cell receptor (TCR)-mediated cytokine synthesis, a common assay of T cell activation by antigen, is modulated by antigen-independent signals provided by various positive or negative costimulator proteins (the latter known as checkpoint inhibitors) expressed by antigen presenting cells, including ECs. We report here that some EC-expressed costimulators also modulate TCR-TEM, but effects differ between TEM and cytokine production and among some T cell types. Blocking EC LFA-3 interactions with T_EM_ CD2 boosts TEM but reduces cytokine production. Blocking EC ICOS-L interactions with T_EM_ CD28 (but not ICOS) reduces both responses but these involve distinct CD28-induced signals. Activated CD4+ T effector cells no longer undergo TCR-TEM. Engagement of T cell CD28 by EC ICOS-L increases TCR-TEM by activated CD8 effectors while engagement of OX40 promotes TCR-TEM by activated CD4 T regs. B7-H3 mostly affects TEM of resting T_EM_ and some checkpoint inhibitors affect cytokine synthesis or TEM depending upon subtype. Our data suggest that blockade or mimicry of costimulators/checkpoint inhibitors *in vivo*, clinically used to modulate immune responses, may act in part by modulating T cell homing.

## Introduction

Circulating leukocytes may be recruited to a peripheral tissue site of inflammation through a well described cascade of events involving chemokines and adhesion molecules expressed by microvascular endothelial cells (ECs) that have been activated by pro-inflammatory cytokines such as IL-1 or TNF (1). Freshly isolated T effector memory cells (T_EM_) can undergo chemokine receptor-induced transendothelial cell migration (CR-TEM) across an endothelial cell monolayer *in vitro* under conditions of flow that mimic the environment of the microvasculature. During this process, T_EM_ initially flatten out, move their microtubular organizing center, granules and mitochondria to a trailing uropod, and then crawl to a site at are near an inter-EC junction where they undergo a form of diapedesis in which the nucleus crosses the EC barrier ahead of the uropod (2, 3). Unlike other leukocytes, T_EM_ alternatively may home to peripheral tissues in response to display of their cognate peptide antigens bound to MHC molecules on the luminal surface of microvascular endothelial cells (ECs) (4), a process we refer to as T cell receptor for antigen (TCR)-mediated homing *in vivo* or TCR-TEM *in vitro*. Morphologically, T_EM_ undergoing TCR-TEM initially round up, forming an immune synapse-like contact with the EC surface. They move their MTOC, granules and mitochondria to the region in contact with the EC surface and then push a blunt protrusion through the EC monolayer in which the MTOC, granules and mitochondria precede the nucleus across the EC monolayer. CD4 T_EM_ but not CD8 T_EM_ must partially degranulate to cross the EC monolayer (3).

TCR-TEM is an important process for the initiation of certain types of immune responses. In a mouse cardiac transplantation model, TCR-mediated homing induced by recognition of luminally displayed antigen was critical for causing T cell-mediated rejection (5). While it is likely that a similar antigen-dependent T cell recruitment mechanism occurs in humans, there are substantial differences between murine and human ECs regarding their capacity for antigen presentation to T cells that must be considered. In mice, microvascular endothelial cells (ECs) basally express class I but generally not class II MHC molecules *in situ*, enabling antigen presentation to CD8 T cells, while human ECs basally express both class I and II MHC molecules, enabling antigen presentation to both CD8 and CD4 T cells (6). There are also significant differences in the expression of costimulatory molecules that bind to and activate auxiliary signals in an antigen-independent manner that modulate the TCR response. The recruitment of T cells in response to antigen in mice depends upon costimulation provided by EC CD80 (B7.1) or CD86 (B7.2) binding to T cell CD28 (7). Human ECs lack both B7.1 and B7.2 (8). However, human ECs do express a number of other costimulatory molecules that can preferentially activate T_EM_ (9). The most potent costimulatory activity for human memory T cells, assessed by boosting antigen-induced cytokine production and proliferation, is LFA-3 (CD58). LFA-3 is a high affinity ligand for T cell CD2 and memory T cells significantly increase their expression of CD2 compared to levels on naïve T cells. The gene for LFA-3 does not exist in mice, and while mice can engage CD2 through CD48, this is a low affinity interaction and cannot functionally replace costimulation by LFA-3. Interestingly, human ECs can express an alternative ligand for CD28, namely ICOS-L (CD275, also known as B7-H2), an interaction that does not occur in mice (10). Other human EC-expressed costimulatory molecules that can boost responses to antigen by memory T cells include 4-1BB-L (CD137L) and OX40L (CD252). Less is known about the functional effects of other EC-expressed costimulators such as Herpesvirus entry mediator (HVEM or CD270) and B7-H3 (CD276) on T cell responses.

Human ECs may also express ligands that inhibit memory T cell activation, including PD-L1 (CD274) and PD-L2 (CD273) which are induced by IFN-γ and engage T cell PD-1 (CD279) (11). These molecules are referred to either as negative costimulators or as checkpoint inhibitors. Human ICOS-L can also bind CTLA-4, an inhibitor of activation of naïve and memory conventional CD4 and CD8 T cells. As a further complication, CTLA-4 engagement can boost the activity of T regulatory cells (T regs) (12). In effector cells, CTLA-4 is induced upon T cell activation and outcompetes CD28 for engagement of B7.1 and B7.2. It is unclear if it can outcompete human CD28 for interactions with ICOS-L. Human ECs also constitutively express PVR (CD155) and nectin-2 (CD112), activating ligands for T cell molecules DNAM-1 (CD226) and Tactile (CD96). These same EC molecules can engage T cell inhibitory receptors TIGIT and PVRIG (all participants in the “DNAM-1 axis” (13)). Thus the effect of a particular costimulatory molecule expressed by ECs may vary with subtypes of T cells that differ in their expression of receptors.

Interestingly, PVR and nectin-2, along with PECAM-1 and CD99, are components of the EC lateral border recycling compartment (LBRC) (14). The LBRC consists of multiple small infoldings of the plasma membrane near or at inter-EC junctions. During leukocyte extravasation, the LBRC is externalized so that the surface proteins found in this compartment line the walls of the EC membrane-lined channels through which leukocytes diapedese. Various LBRC proteins engage specific counter-receptors on the leukocytes that facilitate TEM of neutrophils and monocytes (15). We have previously reported that the EC LBRC, including PECAM-1, CD99, PVR and nectin-2, as well as T cell DNAM-1 and Tactile, are not involved in T_EM_ CR-TEM, but are selectively involved during TCR-TEM of CD4 but not CD8 T_EM_ (16). Thus the positive or negative costimulator effects, if any, of LBRC proteins on diapedesing T cells, is likely to be more evident in CD4 T cell populations.

Historically, both positive and negative co-stimulation are typically defined by their effects upon T cell activation, assessed as proliferation, differentiation or manifestation of effector functions, such as cytokine production or killing. The signaling pathways that are activated by engagement of antigen and costimulators that mediate activation are well described (17). Our prior studies have shown that some of these pathways are involved in TCR-TEM but some, such as calcium release from the endoplasmic reticulum and subsequent activation of plasma membrane calcium release activated channels, actually inhibit this process whereas calcium entry through other TCR-activated plasma membrane calcium channels are required (18). There are also some differences between the signaling pathways used by CD4 and CD8 T_EM_. This raises the prospect that specific costimulators may not only differentially affect T cell cytokine production and TCR-TEM, but that different costimulators may also differentially affect TCR-TEM by CD4 and CD8 T_EM_.

Circulating CD4 T regulatory cells (T regs) also depend upon TCR-mediated antigen recognition and upon costimulation for exerting their functions, but these often differ from the responses of effector T cell populations (19). Because they are selected to recognize self antigens in the thymus, circulating T regs are likely in an activated state under normal conditions (20). Mouse T regs also may be recruited to specific sites using their TCR (4, 21), but the process of TCR-homing or TCR-TEM has not been studied to date with human T regs. Often, these cells are too rare compared to conventional T cells to study their behaviors without ex vivo expansion.

Our prior work has focused on initiation of the immune response which typically involves recruitment of circulating but resting T_EM_ cell populations as well as CD4 T regulatory cells (Tregs). However, as the immune response progresses, antigen activated T effector and regulatory cells are generated within the secondary lymphoid organs, enter the circulation, and are also then recruited to peripheral sites of antigenic challenge. Whether TCR-TEM participates in the recruitment of activated human T effector or regulatory cells to a site of EC antigen presentation is also unknown. Here we report the results of comparing costimulator or checkpoint blockade on TCR-induced cytokine synthesis and TCR-TEM of both resting and activated T cell populations. We find that some EC costimulators can modulate TCR-TEM of resting and activated T_EM_ but that their contributions to this process may differ from those involved in conventional TCR responses. The underappreciated contributions that costimulators and checkpoint inhibitors make to T cell homing may have significant impact on the use of mimetics and inhibitors of these pathways in clinical therapies.

## Materials and methods

### Cells and reagents

All human materials were obtained from de-identified blood or tissue donors under protocols approved by the Yale Human Investigation Committee. Human dermal microvascular endothelial cells (HDMEC) were isolated as described (22, 23). Deidentified and discarded normal human skin was obtained through the Yale Department of Pathology, cut into 3 X 10 cm sections, stretched flat, and sectioned horizontally using a Webster skin graft knife outfitted with a 0.016-in depth gauge and guard to collect the epidermis and superficial layers of the dermis that contain the superficial vascular plexis. After a 40-min incubation in dispase at room temperature, the epidermis was peeled off and cells from both sides of the underlying dermis were gently scraped into RPMI 1640 media and filtered through a 70-µm nylon mesh. The filtrate, containing single cells, was washed once in EGM-2-MV media and plated onto tissue culture plastic pre-coated with 40 µg/ml human plasma fibronectin. HDMECs were allowed to attach approximately 3 h before gentle aspiration (to remove unattached cells) and addition of fresh EGM-2-MV. HDMECs were purified by anti-CD31-biotin miniMACS and serially passaged. CIITA-transduced human dermal microvascular ECs (CIITA HDMEC) were generated using a retroviral vector and characterized as described (24). Prior to flow experiments, CIITA HDMECs were incubated in the presence of 10 ng/ml recombinant human TNF (rhTNFα, R&D Systems) for 20-24 hours to upregulate adhesion molecules, and with 100 ng/ml recombinant TSST-1 (R-TT 606, Toxin Technology, Inc.) for 30 minutes on CIITA HDMECs to allow TCR-mediated activation of all T cells utilizing Vβ2 gene segment to form their TCR, approximately 5-10% of the circulating T cells in most donors. For siRNA knockdown of ICOSL, B7-H3, HVEM, and OX40L, cells were transfected 72 h prior to flow with 10 nM siRNA (ICOSL: Qiagen ICOSLG_5, B7-H3: Ambion s37289, HVEM: Ambion s16701, OX40L: Qiagen TNFSF4_1) as described (16, 25).

Leukapheresis was performed on anonymized healthy volunteer adult donors in the Yale-New Haven Medical Center Blood Bank or purchased from the American Red Cross. The collected PBMCs were enriched by Ficoll-Hypaque density gradient centrifugation prior to cryopreservation of aliquotted cells suspended in fetal calf serum. Total peripheral blood CD4 and CD8 T cells were isolated from the cryopreserved samples by positive selection with CD4 or CD8 Dynabeads magnetic beads and released with Detachabead (Dynal) according to the manufacturer’s protocol. CD4 and CD8 T_EM_ were enriched by depletion of naïve and central memory cells with anti-CCR7 mAb (BioLegend) and pan-mouse IgG beads. Approximately 80-90% (CD4) or 60-70% (CD8) of the initial T cell population as well as essentially all other leukocyte types were removed by these manipulations. The remaining T cells, highly enriched for T_EM_, were cultured in RPMI 1640 medium supplemented with 10% fetal bovine serum, 2 mM glutamine, and penicillin/streptomycin overnight prior to assays. Tregs were isolated by depleting CD127+ cells from a CD4+ positively isolated population, and isolating CD25^high^, CD127- cells by FACS. Isolated CD8 T_EM_ and Treg were activated and expanded by incubating with ImmunoCult T cell activator (CD3, CD28, CD2, StemCell) for 2 d in X-Vivo-15 with 10% FBS, 2 mM glutamine, 1X penicillin, and 100 u/ml IL-2 (Thermo Scientific) and then propagated in the same medium without Immunocult for several weeks.

For blocking antibody experiments, T cells were incubated with anti-CD2 (TS2/18, gift of Timothy Springer), anti-4-1BB (Biolegend 944903), anti-OX40 (R&D AF3388), anti-ICOS (R&D MAB69753), anti-CD28 (Invitrogen 16-0288-81), anti-CTLA-4 (clone BN13, Bio X Cell), anti-PVRIG (clone CHA.7.518-H4)) and anti-TIGIT (clone CPA.9.083-H4) at saturating concentrations for 30 min prior to cytokine and TEM experiments. For PI3K inhibition, T cells were treated with 0.5 µM Omilpalsilib (Selleck Chemicals).

### TEM assays

CIITA HDMEC were grown to confluence on 10 µg/ml human plasma fibronectin-coated 35 mm coverglasses, treated with TNF and loaded with 100 ng/ml TSST-1 as described (24), washed with RPMI/10% FBS, and assembled with a parallel plate flow chamber apparatus (Glycotech) using the 0.01 inch height, 5 mm wide slit gasket provided by the manufacturer. On a 37°C heating surface, freshly isolated CCR7^low^ human CD4 or CD8 T_EM_ or activated/expanded CD8 T_EM_ or Treg (2 x 10^6^ cells/500 µl) or suspended in the same medium were loaded onto the EC monolayer at 0.75 dyne/cm^2^ for 2 minutes, followed by washing with medium only at 1 dyne/cm^2^ for 5, 30, 50 or 60 minutes. Samples were then fixed with 3.7% formaldehyde in PBS, stained with anti-Vβ2TCR mAb (Beckman Coulter), followed by Alexafluor 488 or 546-conjugated goat or donkey anti-mouse IgG, mounted on slides using mounting medium containing DAPI (Prolong Gold, Invitrogen). Alternatively, samples were stained with FITC conjugated anti-Vβ2TCR mAb (Beckman Coulter), AlexaFluor 488-conjugated rabbit anti-FITC, and AlexaFluor 488-conjugated goat anti-rabbit IgG. Samples were then examined by microscopy with a Zeiss Axiovert 200M microscope. A FITC filter was used to detect FITC or Alexafluor 488-stained cells, a TRITC filter was used to detect Alexafluor 546-stained cells, and a DAPI filter used to detect DAPI-stained nuclei. Using a 40X/0.60 korr Ph2 objective, phase contrast optics were used to determine whether T cells were either on top or underneath the HDMEC monolayer. The percentage of transmigrated T cells were calculated for 100 cells per sample by analyzing five groups of 20 cells each, calculating the percentage for each group, and calculating the mean and s.e.m. for the groups. For total adhesion, T cells in ten fields using a 10X objective were counted for each sample. For antigen-induced adhesion, the percentage of Vβ2TCR+ cells from a total of more than 200 total cells counted was calculated for each sample. This percentage was divided by the percentage of Vβ2TCR+ cells of the input as determined by FACS to obtain the fold enrichment.

#### Cytokine expression assay

200,000 T cells were incubated with TNF-activated, TSST-1 preloaded CIITA HDMEC in C24 wells for 3 h. Samples were then processed for RNA preparation (RNeasy Mini Kit, Qiagen) and cDNA (High Capacity cDNA Reverse Transcriptase Kit, Applied Biosystems). Quantitative real time PCR using TaqMan probes for human IFN-γ, IL-2, IL-10 and CD3e and TaqMan Gene Expression Master Mix (Applied Biosystems) were performed in a C1000 Touch Thermal Cycler CFX96 Real-Time System (Biorad). Threshold cycles (C_T_) of cytokine were normalized with C_T_ of CD3e to compute the absolute levels of cytokine expression, and absolute levels of cytokine expression of various treated conditions were divided by the absolute values of control to obtain relative values compared to control.

#### Confocal microscopy

To visualize MTOC of activated/expanded Vβ2^+^ CD8 T_EM_ and Treg transmigrating on TNF-activated CIITA HDMEC plus TSST-1, samples were stained with anti-Vβ2TCR mAb (Beckman Coulter), Alexa Fluor 546-conjugated donkey anti-mouse IgG (Invitrogen), permeabilized with 0.5% Triton/PBS, re-fixed with methanol/acetone (50/50), stained with rabbit anti-γ-tubulin (Sigma), Alexa Fluor 647-conjugated donkey anti-rabbit IgG and Alexa Fluor 488-conjugated phalloidin, and mounted on slides using mounting medium containing DAPI (Prolong Gold, Invitrogen).

Images of single T cells on the EC apical surface were captured with a Leica TCS SP5 Spectral Confocal Microscope, 405UV using a 63X oil immersion objective and sequential scanning with 405 Diode, argon and He/Ne laser excitation lines of 405 nm, 488 nm, 543 nm, and 633 nm. Six Z slices were captured encompassing the entire T cell starting from the EC interface.

#### Treg suppression assay

250,000 PBMC stained with Cell Trace Violet in the present or absence of CD3 activating Ab OKT3 were incubated with 100,000, 50,000, 25,000, 12,500 or 0 activated/expanded CD4 Treg for 5 d, stained with CD8-APC, and analyzed by FACS.

#### Statistical analyses

For experiments in which more than two groups were compared, statistical significance was determined by one-way ANOVA using a 95% confidence interval and the Tukey post-test (Prism 6.0 for Macintosh). Statistical error is expressed as s.e.m. For experiments in which two groups were compared, a t-test was used. P values are designated as: *, p<0.05; **, p<0.01; ***, p<0.001; ****, p<0.0001.

## Results

### Experimental approach

We use blocking Abs to T cell receptors of costimulation molecules or siRNA knockdown of EC expressed costimulatory molecules to examine the contributions of EC/T cell costimulatory molecule interactions on cytokine expression and TCR-TEM. For these experiments, we use TNF-treated CIITA-HDMEC loaded with superantigen TSST-1. TSST-1 selectively activates only T cells that contain Vβ2 segments in their TCR, normally present in about 5-10% of the circulating T cell population, which can be identified by immunofluorescence staining with an anti-Vβ2TCR mAb. TSST-1 signals through the TCR and serves as a surrogate antigen for human T cell populations that express millions of clonally distributed distinct TCRs. Even after immunization, the percent of T_EM_ or T regs specific for a particular antigen is generally less than 1 in 10,000. Activation of the Vβ2TCR+ T cells induces cytokine mRNA expression more than 1000-fold after 3 h as measured by qRT-PCR normalized to CD3ε expression (18).

### TCR-TEM of resting CD4 T_EM_

**Figure 1:**
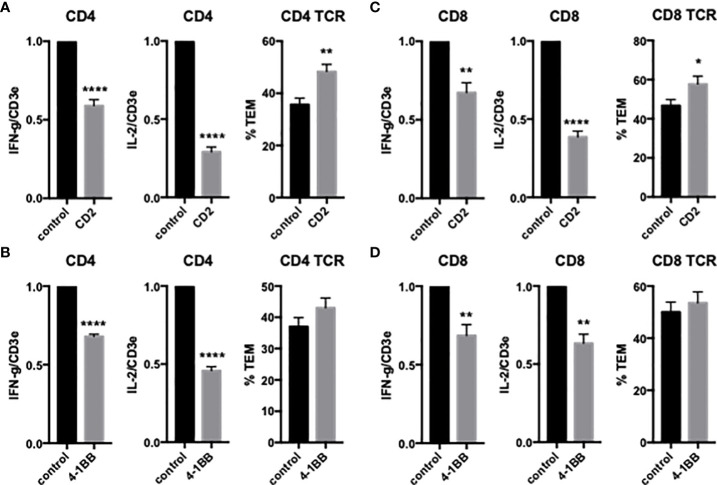
Effects of CD2 and 4-1BB costimulatory molecules on cytokine expression and TCR-TEM of freshly isolated CD4 and CD8 T_EM_. Blocking Abs to T cell CD2 **(A, C)** or 4-1BB **(B, D)** were added to T cells 30 min prior to addition to CIITA-transduced HDMEC activated with TNF 24 h, overlaid with TSST-1 30 min and assayed for cytokine expression by QRTPCR after 3 h (left graphs) and TCR-TEM after 50 min (CD4, A, B) and 30 min (CD8, C, D) flow by staining fixed samples for Vβ2TCR and analyzing TEM of Vβ2TCR+ cells identified by fluorescence and phase contrast microscopy to determine whether those cells have transmigrated or not (right graphs). *p<0.05; **p<0.01; ****p<0.0001.

We first examined the effects of EC LFA3/T cell CD2, EC 4-1BBL/T cell 4-1BB and EC OX40L/T cell OX40 on freshly isolated CD4 T_EM_ cytokine expression and TCR-TEM. Blocking CD2 and 4-1BB significantly reduces cytokine expression, but surprisingly CD2 blockade enhances TCR-TEM whereas blocking 4-1BB has no effect on TCR-TEM (**Figure 1**). OX40 blockade has no effect on either cytokine expression or TCR-TEM (data not shown).

**Figure 2:**
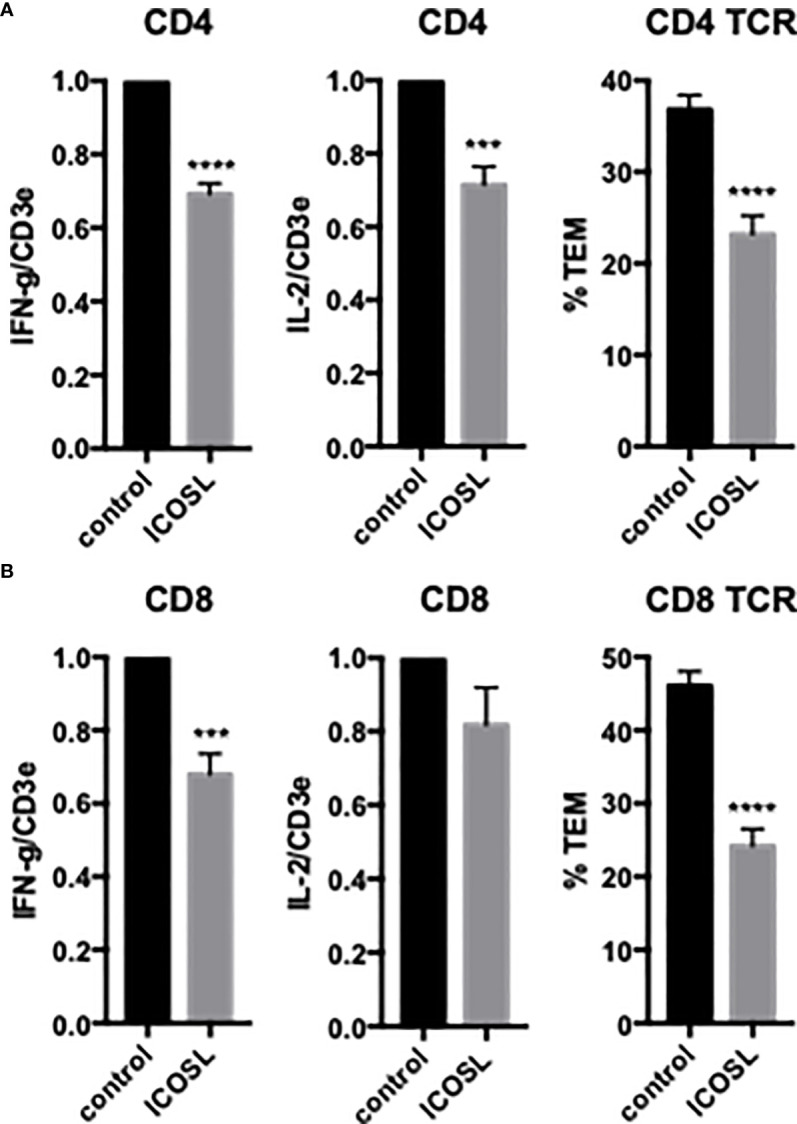
Effects of EC ICOSL on cytokine expression and TCR-TEM of CD4 and CD8 T_EM_. CD4 **(A)** and CD8 **(B)** T_EM_ were assayed as in **Figure 1** on CIITA HDMEC treated with control or ICOSL siRNA. ***p<0.001; ****p<0.0001.

**Figure 3:**
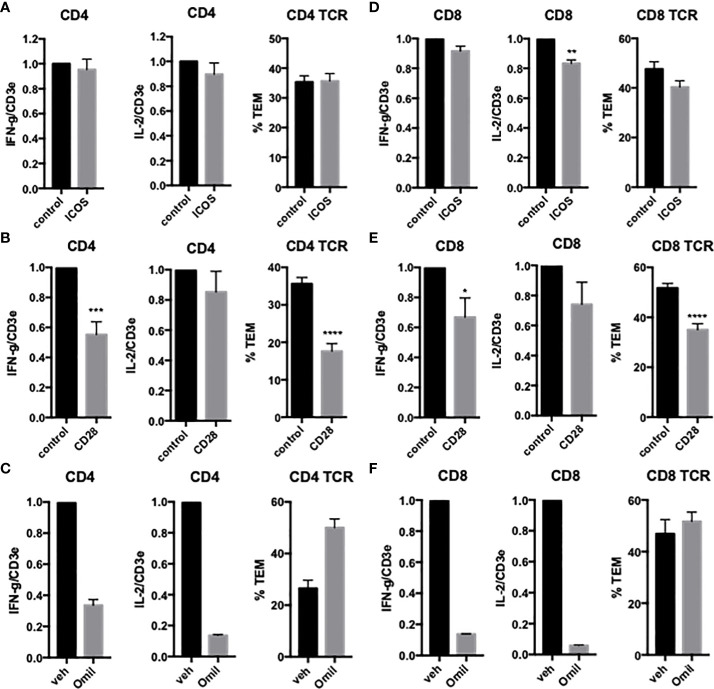
Effects of T cell CD28 and ICOS on cytokine expression and TCR-TEM of CD4 and CD8 T_EM_. CD4 **(A, B)** and CD8 **(D, E)** T_EM_ treated with ICOS blocking Ab **(A, D)** or CD28 blocking Ab **(B, E)** assayed as in **Figure 1**. Cytokine expression and TCR-TEM of CD4 **(C)** and CD8 **(F)** T_EM_ cells treated with Omilpalisib (Omil) assayed as in **Figure 1**. *p<0.05; **p<0.01; ***p<0.001; ****p<0.0001.

To examine the effects of EC ICOSL on freshly isolated CD4 T_EM_ TCR-TEM and cytokine expression, we treated HDMEC with siRNA to ICOSL, resulting in approximately 90% knockdown (data not shown). Knockdown of HDMEC ICOS-L expression decreased both cytokine expression and TCR-TEM (**Figure 2**). Since human ICOSL has two costimulatory receptors on T cells, namely ICOS and CD28, we interrogated both using blocking Abs. Blocking CD4 T_EM_ ICOS has no effect on cytokine expression or TCR-TEM, but blocking CD28 reduces cytokine expression at a low but statistically significant level and markedly reduces TCR-TEM (**Figure 3**). Since human ECs lack B7.1 and B7.2, these responses likely result from EC ICOS-L engagement of T cell CD28. Since CD28 is known to signal through activation of phosphotidyl inositol 3 kinase (PI3K), we tested the effects of a PI3K inhibitor (Omilpalisib) on cytokine expression and TCR-TEM. PI3K inhibition decreased cytokine expression, but increased CD4 T_EM_ TCR-TEM and had no effect on CR TEM (**Figure 3** and not shown). These data suggest that CD28 signals that affect TCR-TEM likely utilize pathways other than PI3K activation. Interestingly, we observed no effect in TCR-TEM assays of CD4 T_EM_ by blocking CTLA-4 and a small increase in cytokine production that did not reach statistical significance (**Supplemental Figure 1**).

**Figure 4:**
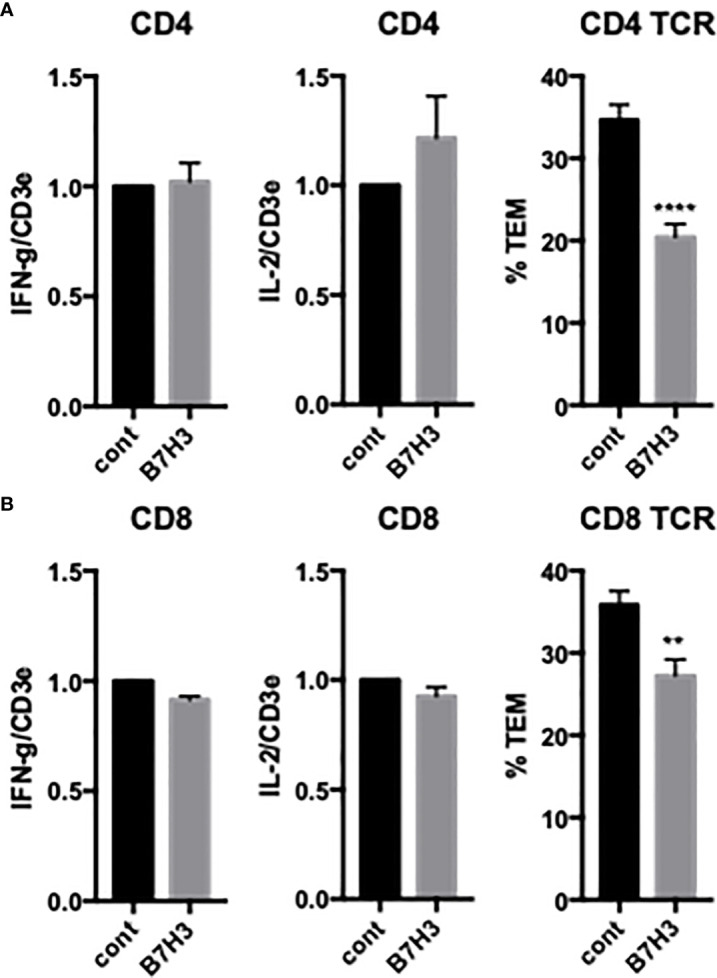
Effects of EC B7H3 on cytokine expression and TCR-TEM of CD4 and CD8 T_EM_. Cytokine expression and TCR-TEM of CD4 **(A)** and CD8 **(B)** T_EM_ on CIITA-transduced HDMEC treated with control or B7H3 siRNA, activated with TNF 24 h, and overlaid with TSST-1 30 min and assayed as described in **Figure 1**. **p<0.01; ***p<0.0001.

HDMECs also express other potential costimulatory ligands, including HVEM and B7-H3. Knockdown of HDMEC HVEM had no effect on either cytokine expression or TCR-TEM of CD4 T_EM_ (data not shown), but knockdown of B7-H3 selectively inhibited TCR-TEM (**Figure 4**). The T cell receptor for EC B7-H3 is unknown.

**Figure 5:**
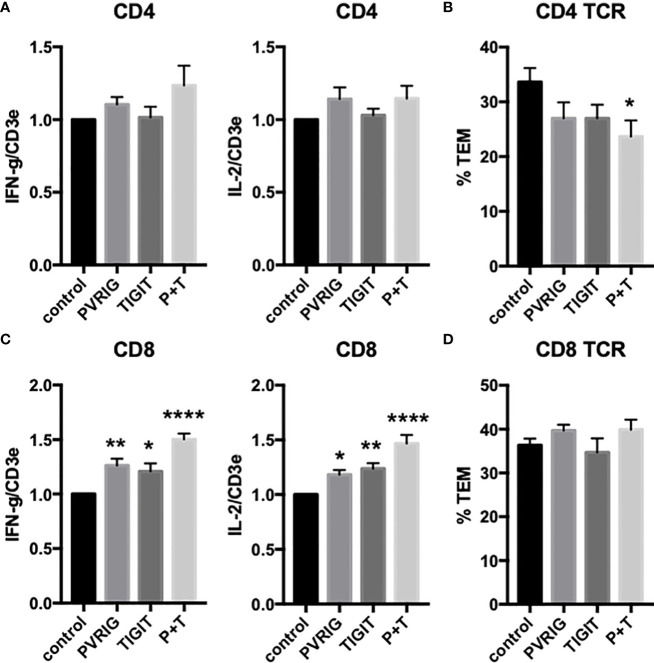
Effects of T cell TIGIT and PVRIG on cytokine expression and TCR-TEM of CD4 T_EM_ and CD8 T_EM_. CD4 **(A, B)** and CD8 **(C, D)** T_EM_ treated with TIGIT and/or PVRIG blocking Abs and assayed as in **Figure 1**. *p<0.05; **p<0.01; ****p<0.0001.

T cells also express PVRIG and TIGIT, known coinhibitory receptors in the “DNAM-1 axis” that interact with PVR (CD155) and nectin-2 (CD112). We have previously reported that EC PVR and nectin-2, as well as CD4 T_EM_ DNAM-1 (CD226) and Tactile (CD96), contribute to CD4 T_EM_ TCR-TEM (16, 26). Since these molecules (as well as PECAM-1 and CD99, which also contribute to CD4 T_EM_ TCR-TEM) reside in the LBRC, we interpret these results to indicate that mobilization of the LBRC is involved in TCR-TEM of CD4 T_EM_. Inhibiting PVRIG or TIGIT by blocking antibodies had no significant affect on cytokine expression but somewhat decreased TCR-TEM of CD4 T_EM_ (**Figure 5**). These observations suggest that the DNAM axis plays a role in CD4 T_EM_ TCR-TEM, but there is considerable redundancy since activators and inhibitors of the DNAM axis appear to exert similar effects

### TCR-TEM of resting CD8 T_EM_

We next investigated freshly isolated CD8 T_EM_ using the same assays outlined above for CD4 T_EM._ Like CD4, blocking CD8 T_EM_ CD2 and 4-1BB inhibited cytokine expression but did not inhibit TCR-TEM and blocking OX40 had no effect (**Figure 1** and data not shown). Knockdown of EC ICOSL, blocking T cell ICOS and CD28 also had very similar effects on CD8 as compared to CD4 T_EM_ (**Figures 2**, **3**). However, while PI3K inhibitor similarly decreased cytokine expression, it had no effect on TCR-TEM of CD8 T_EM_. Knockdown of EC HVEM had no effect on either cytokine production or TCR-TEM, while knockdown of EC B7-H3 reduced TCR-TEM (**Figure 4** and data not shown). In contrast to CD4 T_EM_, blocking CD8 T_EM_ PVRIG and TIGIT boosted cytokine expression but had no effect on TCR-TEM (**Figure 5**).

### TCR-TEM of “resting” CD4 T regs

We were also interested in investigating another subset of T cells, namely freshly isolated “resting” CD4+, CD127-, CD25^high^ regulatory T cells (Tregs). As noted in the Introduction, it is likely that these cells are chronically stimulated by low affinity TCR signals through interactions with self-peptides displayed on self-class II HLA molecules expressed basally on human ECs. Initial assays of freshly isolated Tregs suggested that they also displayed the hallmarks of TCR-TEM (i.e., slower kinetics compared to CR-TEM, MTOC polarization). However, we were unable to isolate sufficient “resting” T regs to examine the effects of various costimulator interactions.

### TCR-TEM of activated CD4 T effector cells

While the behaviors of resting but circulating T_EM_ serve as a model for initiating immune responses, ongoing responses involve activated and expanded effector or regulatory cells. To model this process, we polyclonally activated and expanded CD4 and CD8 T_EM_ as well as CD4 Tregs (isolated as CD4+, CD127-, CD25^high^). Initial characterization indicated that activated and expanded CD4 T_EM_ appear to no longer undergo TEM in response to TCR signals (**Supplemental Figure 2**). Our prior work had indicated that in addition to use of the LBRC, CD4 T_EM_ required engagement of fractalkine expressed on ECs to undergo TCR-TEM. Freshly isolated CD4 T_EM_ and CD8 T_EM_ express fractalkine receptor, but fractalkine receptor is not needed for CD8 T_EM_ TCR-TEM (**Supplemental Figure 2**). Fractalkine receptor is lost upon polyclonal expansion of both CD4 and CD8 T_EM_. Reintroduction of the fractalkine receptor by transduction restored the process of TCR-TEM in expanded CD4 T_EM_ and had no effect on CD8 T_EM_ TCR-TEM (**Supplemental Figure 2**). Since our focus is on modeling the behavior of expanded T cells *in vivo*, we did not further investigate activated/expanded CD4 T_EM_.

### TCR-TEM of activated CD8 effector T cells

**Figure 6:**
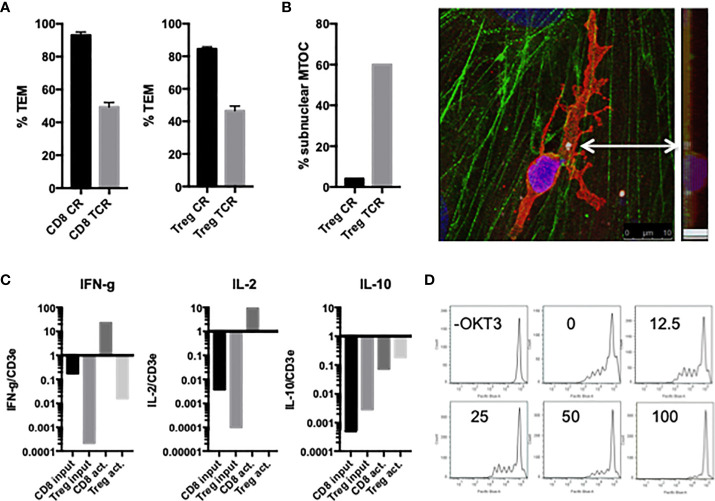
Characterization of activated/expanded CD8 T_EM_ and CD4 Treg. **(A)** TEM of activated and expanded CD8 T_EM_ and CD4 Treg whether activated by superantigen presented by EC (TCR) or not (CR) after 60 min flow. **(B)** MTOC polarizes between the activated Treg cell nucleus and EC surface at early time points (left graph), and then precedes the nucleus during diapedesis (image of confocal slices merged and viewed from on top and at a 90 degree angle). Samples stained for VB2TCR (red) gamma tubulin (white, indicated by arrow) phalloidin (green) and DAPI (blue). **(C)** Relative cytokine expression levels in activated/expanded CD8 T_EM_ and CD4 Treg. **(D)** Treg suppression assay. 250,000 PBMC stained with Cell Trace Violet in the present or absence of CD3activating Ab OKT3 were incubated with 100,000, 50,000, 25,000, 12,500 or 0 activated/expanded CD4 Treg for 5 d, stained with CD8-APC, and analyzed by FACS. Shown are histograms of CD8+ cells.

Similar to freshly isolated CD4 and CD8 T_EM_, activated and expanded CD8 T_EM_ transmigrated slower upon TCR activation compared to non-activated cells (**Figure 6**), and polarize the MTOC to a position between the T cell nucleus and EC apical surface upon contact with antigen-presenting HDMEC which then precedes the nucleus during diapedesis, i.e., hallmarks of TCR-TEM (data not shown). Similar to freshly isolated T_EM_, cytokine expression profile favored IFN-γ and IL-2 and increased markedly upon activation (**Figure 6**).

**Figure 7:**
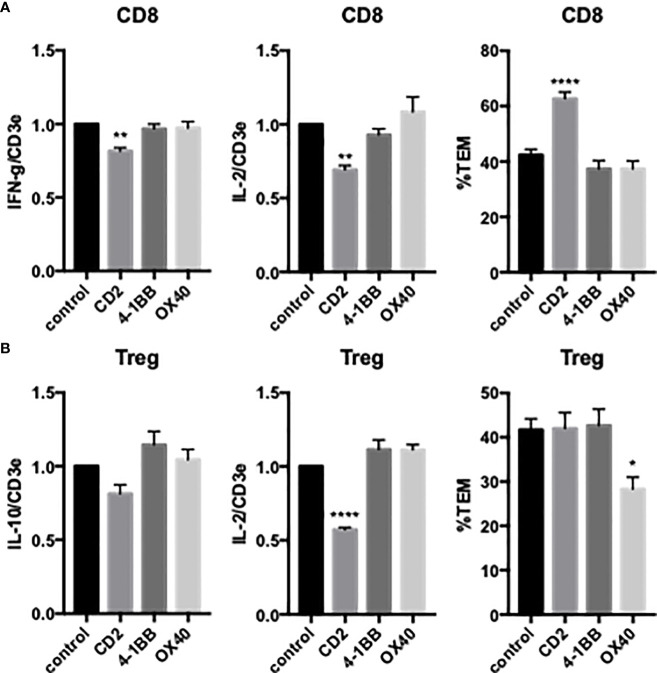
Effects of T cell CD2, 4-1BB and OX40 on cytokine expression and TCR-TEM of activated/expanded CD8 T_EM_ and CD4 Treg. Activated/expanded CD8 T_EM_ **(A)** and CD4 Treg **(B)** were treated with blocking antibodies to CD2, 4-1BB, or OX40 and assayed for cytokine expression after 3 h (IFN-γ and IL-2 for CD8, IL-10 and IL-2 for Treg, left and middle graphs) and TCR-TEM after 60 min flow (right graphs). *p<0.05; **p<0.01; ****p<0.0001.

**Figure 8:**
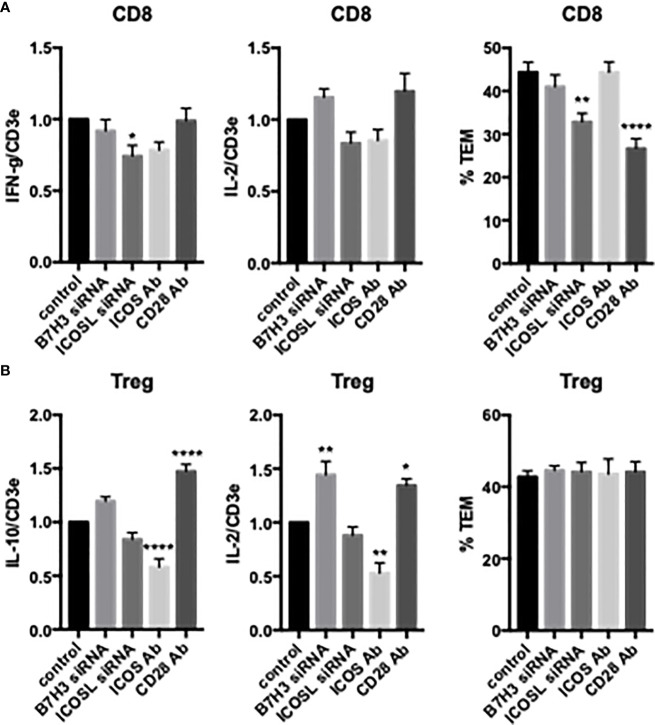
Effects of EC B7H3 and ICOSL knockdown by siRNA and T cell ICOS and CD28 blocking Abs on cytokine expression and TCR-TEM of activated/expanded CD8 T_EM_ and Treg. Cytokine expression (left and middle graphs) and TCR-TEM (right graphs) of activated/expanded CD8 T_EM_ **(A)** and Treg **(B)** on CIITA HDMEC transfected with B7H3 siRNA or ICOSL siRNA and after pretreatment of T cells with ICOS blocking Ab or CD28 blocking Ab. *p<0.05; **p<0.01; ****p<.0001.

We next examined the effects of costimulators on cytokine expression and TCR-TEM of activated and expanded CD8 T_EM_. CD2 blocking Ab modestly reduced cytokine expression of activated and expanded CD8 and also markedly increased CD8 TCR-TEM, but neither 4-1BB nor OX40 blocking Abs affected cytokine expression or TCR-TEM (**Figure 7**). Knockdown of EC ICOS-L and blocking activated/expanded CD8 T_EM_ CD28 inhibited TCR-TEM, but had minimal to no effect on cytokine expression (**Figure 8**). There were no significant effects observed by blocking CTLA-4 (**Supplemental Figure 3**). EC knockdown of HVEM or B7-H3 had no effect on cytokine expression or TCR-TEM by activated and expanded CD8 T_EM_ (**Figure 8** and not shown).

### TCR-TEM of activated CD4 T regs

Activated and expanded Treg also undergo TCR-TEM as judged by the same morphological criteria mentioned previously. That is, activated and expanded Treg transmigrated slower upon TCR activation compared to non-activated cells (**Figure 6**), and polarize the MTOC to a position between the T cell nucleus and EC apical surface upon contact with antigen-presenting HDMEC which then precedes the nucleus during diapedesis, i.e., hallmarks of TCR-TEM (**Figure 6**). Cytokine expression profile of activated and expanded Tregs favored IL-2 and IL-10: IFN-γ was more than 1000-fold lower in Treg as compared to CD8 T_EM_, but IL-10 was about 10-fold higher in Treg as compared to CD8 T_EM_. However, they all increased markedly upon activation (**Figure 6**). Since the levels of IFN-γ were too low to measure accurately by qRT-PCR, we instead measured levels of IL-10 in lieu of IFN-γ for Treg. Activated and expanded Treg also retain the capacity to suppress T_EM_ proliferation (**Figure 6**).

Activated/expanded Tregs responded in some cases similarly but also differently to blocking costimulatory molecules. CD2 blocking Ab inhibited cytokine expression but had no effect on TCR-TEM. However, blocking OX40 inhibited activated/expanded Treg TCR-TEM (**Figure 7**). Cytokine expression was reduced by ICOS blocking Ab, but actually enhanced by EC knockdown of B7-H3 and blocking Treg CD28, while none of these treatments affected TCR-TEM (**Figure 8**).

## Discussion

Experiments in mice have shown that antigen-specific circulating T_EM_ may home to specific tissues based upon TCR engagement of cognate antigens displayed by microvascular ECs (21, 27) and that this process may be required to initiate allograft rejection (5). Our prior studies have shown that human CD4 and CD8 T_EM_ also can use their TCR to undergo TEM *in vitro*, a process we called TCR-TEM (3, 16, 18, 24–26, 28). Various positive costimulatory molecules can provide activating or inhibitory signals that complement those generated by engagement of the TCR in CD4 and CD8 T_EM_ as well as in CD4 T regs, affecting cytokine expression, proliferation, differentiation and survival. T cell receptors for specific costimulator molecules vary with the differentiation state of the T cell, consistent with the finding that T_EM_ preferentially interact with ECs related to the costimulatory molecules expressed by this cell type. Our key findings, summarized in **Table 1**, are that preventing engagement of T_EM_ CD2 by the EC costimulator LFA-3 reduces cytokine expression as previously reported (29), but appears to increase TCR-TEM of both resting CD4 and CD8 T_EM_. In contrast, blocking the EC costimulatory ICOS-L binding to T cell CD28 (and not ICOS), an interaction which occurs in humans but not mice (10), reduces both cytokine synthesis as well as TCR-TEM. Blocking CTLA-4 appeared to have little effect upon interactions of T cells with ECs, presumably mediated by ICOS-L. However, the effect on cytokine synthesis can be selectively blocked by a pharmacological inhibitor that has no effect on TCR-TEM, suggesting that different CD28-intiated responses are involved. EC B7H3, the receptor for which on T cells is unknown, also contributes to both CD4 and CD8 T_EM_ recruitment, although the effects are relatively greater with CD4 T_EM_. Blocking the inhibitory signals provided by PVRIG and TIGIT increased cytokine production as expected, but selectively decreased TCR-TEM of CD4 T_EM_ only to a minor degree and did not affect CD8 T_EM_. While circulating T_EM_ may initiate responses, once begun, activated T effector cells formed in the secondary lymphoid organs can also home to sites of inflammation. Interestingly, activated CD4 T effector cells lose the capacity to use TCR signaling for initiating TEM, a finding likely related to downregulation of fractalkine receptor, which like the use of LBRC proteins, is required for TCR-TEM of CD4 T_EM_ but does not participate in TCR-TEM of CD8 T_EM_ (26). However, both polyclonally expanded CD8 T_EM_ and polyclonally expanded CD4 Tregs have the capacity to undergo TCR-TEM. Blocking T cell CD28 engagement by EC ICOS-L reduces TCR-TEM by activated/expanded CD8 T_EM_, but are no longer influenced by EC B7H3. Activated and expanded Treg do not depend on CD28/ICOSL interactions or EC B7H3 for TCR-TEM, but rather utilize EC OX40L/Treg OX40.

**Table 1:** Summary of costimulatory molecule blocking effects on cytokine expression and TCR-TEM. +, -, 0 denote positive, negative, and no contribution, respectively. The number of symbols refers to significance as displayed in the figures.

		Freshly isolated				Activated/expanded			
		CD4 T_EM_		CD8 T_EM_		CD8 T_EM_		Treg	
EC	T cell	cyto	TEM	cyto	TEM	cyto	TEM	cyto	TEM
LFA-3	CD2	----	++	----	+	--	++++	----	0
4-1BBL	4-1BB	----	0	--	0	0	0	0	0
OX40L	OX40	0	0	0	0	0	0	0	–
ICOSL	ICOS	0	0	--	0	0	0	----	0
ICOSL	CD28	----	----	–	----	–	-----	++++	0
B7H3	?	0	----	0	--	0	0	++	0

It is perhaps surprising that costimulator requirements for CD4 and CD8 TCR-TEM are so similar. We have previously documented the many differences between CD4 and CD8 TCR-TEM, including T cell granule exocytosis and EC LBRC (both required for CD4, not CD8) (26). Recently we found that CD4 but not CD8 also require TCR-activated TRPV1 and L-type Ca_v_ calcium channels. However, both require pannexin-1/P2X receptors, the first example of a potential therapeutic target that would affect both CD4 and CD8 TCR-TEM (18). CD28 is now another potential target albeit the human EC costimulatory involved appears to be ICOS-L rather than B7.1 or B7.2. An anti-CD28 Ab (FK734) that stimulates T cell proliferation and cytokine expression *in vitro*, reduced recruitment of adoptively transferred human T cells in a human immune system mouse model of human skin transplant rejection (30).

T cell receptors for negative costimulators and their ligands, often referred to as checkpoint inhibitors, are now established targets for therapeutics treating cancer. These include T cell CTLA-4, PD-1 and more recently TIGIT and PVRIG. The rationale is that relieving the inhibitory signal can rejuvenate an inherent anti-cancer response. Although initially thought to be an effect occurring within the tumor itself, a recent study shows that these agents most likely act within secondary lymphoid organs on stem-like T cells to generate effector T cells (31). The effector cells that are generated must then enter the circulation and home to the tumor, crossing the ECs of the tumor vasculature. We therefore investigated the effects of these reagents on T cell recruitment and cytokine expression in the context of T cell interacting with EC. Blocking CTLA-4 and PD-1 had no effect in our assays, and while blocking TIGIT and PVRIG had the expected positive effect on cytokine expression, these agents marginally reduced TCR-TEM of CD4 T cells, while not affecting TCR-TEM of CD8 T_EM_ cells. While the minor effect on CD4 T cell migration will not necessarily translate to inhibition of T cell migration *in-vivo*, we speculate that this action has less to do with blocking signaling from the receptors, but rather potentially reflect the adhesive interaction of these receptors with EC ligands, namely PVR and nectin-2, that are components of the LBRC. As we have described previously, CD4 but not CD8 T_EM_ similarly rely on T cell Tactile (CD96) and DNAM-1 (CD226) interacting with the LBRC components for TCR-TEM. Mobilization of the LBRC includes the recruitment of multiple EC proteins (including CD31, CD99, CD112 and CD155) to the inter- or intracellular channel used by leukocytes to facilitate diapedesis (14). Perhaps TIGIT and PVRIG also bind to components of the LBRC and facilitate TCR-TEM of CD4 T_EM_ in a similar manner.

The use of the LBRC is one of several significant differences between TCR-TEM of CD4 and CD8 T_EM._ The other is that CD4 T_EM_ also require signaling from EC fractalkine to undergo this process. Interestingly, activated and expanded CD4 T_EM_ lose expression of the fractalkine receptor. This may explain why these cells appear incapable of undergoing TCR-TEM. Although we could restore TCR-TEM by transducing expression of fractalkine receptor in these cells, this approach was not relevant for the study of endogenous T cell behaviors and although it may be important for adoptive T cell therapies, it was not further studied in the current investigation.

This is the first report describing antigen-driven TEM of human Tregs. These cells are relatively uncommon within the circulation which has previously limited our ability to isolate and study CD4+ CD25^high^ CD127- T cells in our standard TEM assays. We had previously observed that freshly isolated Vβ2+ T regs do undergo TEM in response to TSST-1 in a manner that morphologically fit the description of TCR-TEM, i.e. the cells rounded up and the MTOC preceded the nucleus across the EC monolayer (unpublished observations). By developing populations of polyclonally activated and expanded T regs, we now can further analyze their TEM behaviors. Unlike CD4 but like CD8 T_EM_, activated/expanded CD4 T reg TCR-TEM does not require EC LBRC or fractalkine (data not shown). The sole requirement for OX40 in Treg but not CD4 or CD8 T_EM_ TCR-TEM indicates once again that different T cell subsets have various molecular requirements for traversing the EC monolayer in response to antigen. These differences could be a basis for targeted therapy.

While the focus of this investigation has been to use *in vitro* assays of human T cell TCR-TEM to investigate the roles of positive and negative costimulators in the context of normal, endogenous adaptive immune responses, our findings may have implications for some T cell-based therapeutics. Adoptive immunotherapies with ex vivo expanded T cells or chimeric antigen receptor T cells utilize populations capable of TCR-homing and that express endogenous receptors of co-stimulators. Since these cells must get into tissues, costimulatory interactions may influence effectiveness. Furthermore, second generation chimeric antigen receptors include signaling domains from T cell molecules such as CD28 or 4-1BB. The domains selected are typically based on effects of the endogenous molecule upon activation responses. Our findings with pharmacological blockade of CD28 signaling suggest that the relevant signaling domains for conventional activation and for TCR-homing may not be the same.

In summary, the present study has revealed that EC costimulators can modulate the TCR signaling pathways that result in TCR-based homing. The functional effects of costimulation in this context differs from that observed in other T cell assays and raises both new opportunities and cautions for use of costimulatory and checkpoint inhibitor blockades in immunotherapies.

## Data availability statement

The raw data supporting the conclusions of this article will be made available by the authors, without undue reservation.

## Author contributions

TM conceived and designed the work, acquired, analyzed and interpreted data, and drafted and revised the paper. VW acquired and analyzed data and revised the paper. JP interpreted data, provided key intellectual input, drafted and revised the paper. All authors contributed to the article and approved the submitted version.

## Funding

R21AI154187 NIH-NIAID JP, TM, co-PIs.

## Conflict of interest

The authors declare that the research was conducted in the absence of any commercial or financial relationships that could be construed as a potential conflict of interest.

## Publisher’s note

All claims expressed in this article are solely those of the authors and do not necessarily represent those of their affiliated organizations, or those of the publisher, the editors and the reviewers. Any product that may be evaluated in this article, or claim that may be made by its manufacturer, is not guaranteed or endorsed by the publisher.
