# Supplementary figures and images for: Costimulators expressed on human endothelial cells modulate antigen-dependent recruitment of circulating T lymphocytes
Source: Front Immunol. 2022 Oct 6;13:1016361. doi: 10.3389/fimmu.2022.1016361 (PMC9582530; doi:10.3389/fimmu.2022.1016361)

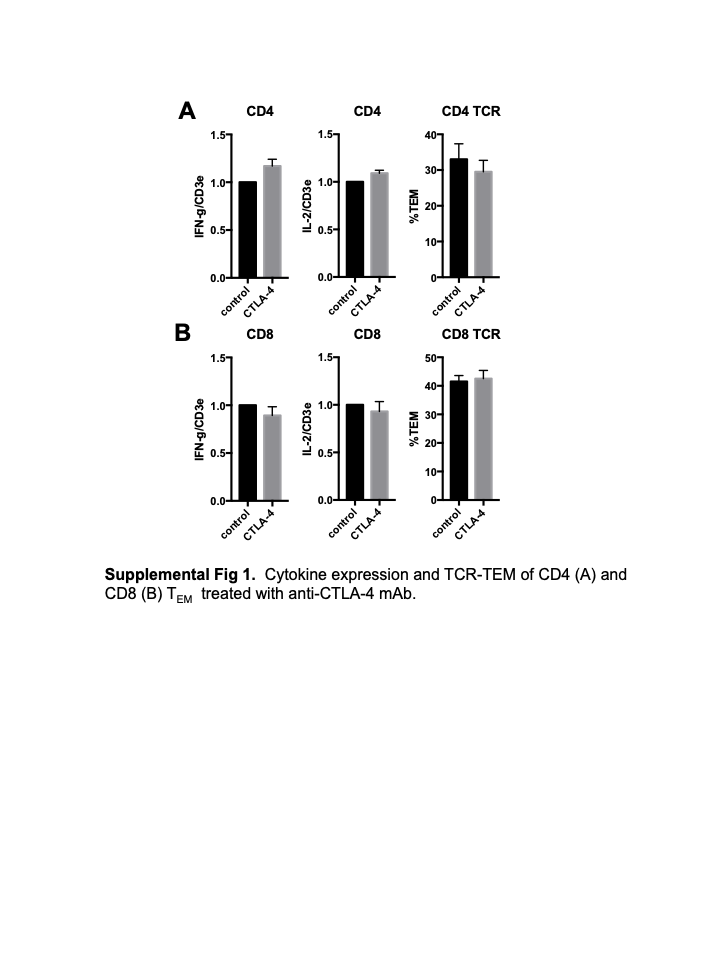

Supplement: Supplementary file 1 [file Image_1.tiff]

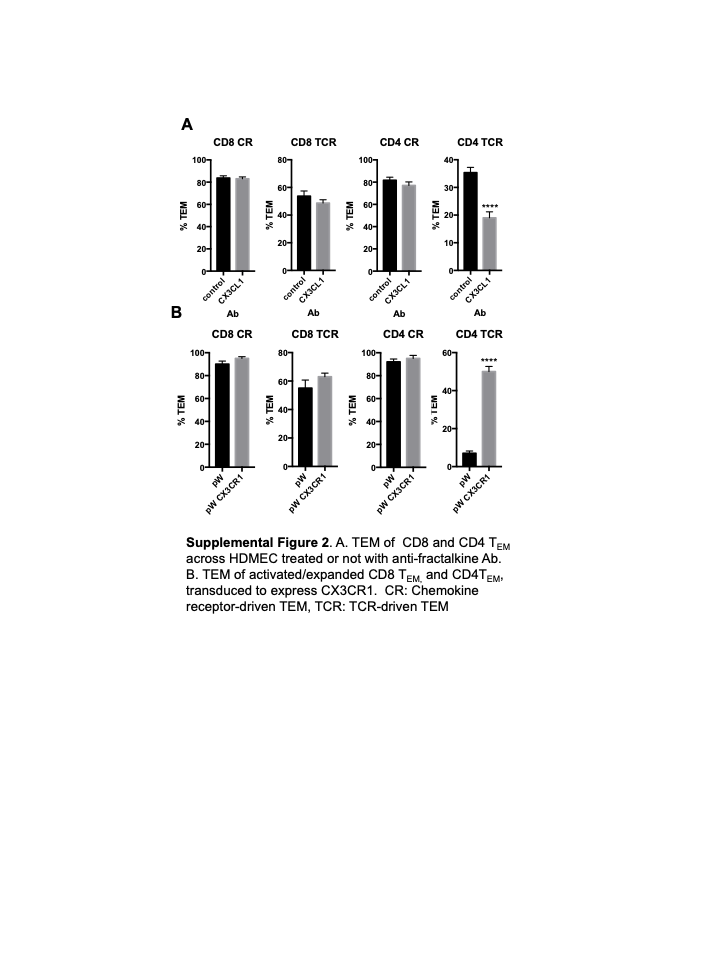

Supplement: Supplementary file 2 [file Image_2.tiff]

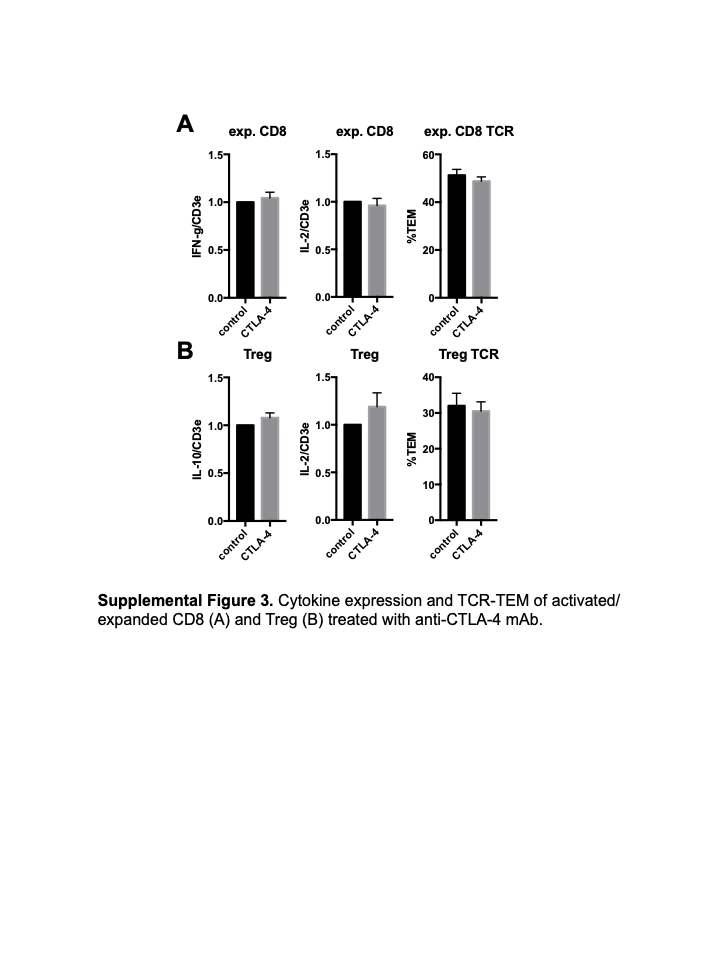

Supplement: Supplementary file 3 [file Image_3.tiff]
